# Selectivity of Enzymatic Conversion of Oligonucleotide Probes during Nucleotide Polymorphism Analysis of DNA

**Published:** 2010-04

**Authors:** O.A. Vinogradova, D.V. Pyshnyi

**Affiliations:** Institute of Chemical Biology and Fundamental Medicine, Siberian Division, Russian Academy of Sciences

**Keywords:** DNA complexes, mismatch, selectivity, DNA ligase, DNA polymerase, modified oligonucleotide probes

## Abstract

The analysis of DNA nucleotide polymorphisms is one of the main goals of DNA diagnostics. DNA–dependent enzymes (DNA polymerases and DNA ligases) are widely used to enhance the sensitivity and reliability of systems intended for the detection of point mutations in genetic material. In this article, we have summarized the data on the selectiveness of DNA–dependent enzymes and on the structural factors in enzymes and DNA which influence the effectiveness of mismatch discrimination during enzymatic conversion of oligonucleotide probes on a DNA template. The data presented characterize the sensitivity of a series of DNA–dependent enzymes that are widely used in the detection of noncomplementary base pairs in nucleic acid substrate complexes. We have analyzed the spatial properties of the enzyme–substrate complexes. These properties are vital for the enzymatic reaction and the recognition of perfect DNA–substrates. We also discuss relevant approaches to increasing the selectivity of enzyme–dependent reactions. These approaches involve the use of modified oligonucleotide probes which “disturb” the native structure of the DNA–substrate complexes.

## INTRODUCTION

Single nucleotide polymorphism (SNP) is the most common form of genetic variations in the genome. Currently, the number of known single nucleotide mutations in the human genome is in excess of 9 million [1]. Such mutations are often important genetic markers that can determine the phenotypic and physiological traits of an individual and are also the molecular basis of certain diseases.

**Fig. 1:**
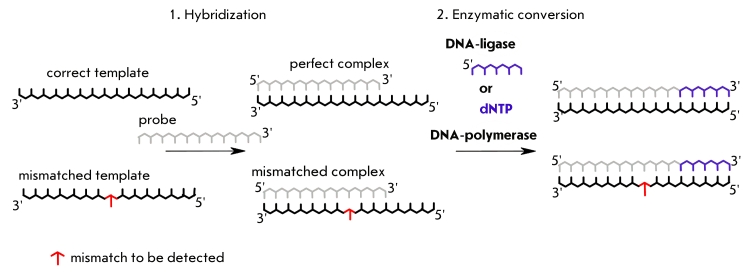
Stages of DNA hybridization analysis using oligonucleotide probes and DNA-dependent enzymes

Detection of single nucleotide substitutions in nucleic acids (NA) using the most effective and simple methods which yield reproducible results is a problem of much practical interest. The development of methods for detecting point mutations which use oligonucleotide probes specific to complementary regions of NA–targets has led to a whole range of approaches based on the use of DNA–dependent enzymes, most often DNA–polymerases [2, 3] and/or DNA–ligases [4, 5]. Currently, methods for detecting single nucleotide substitutions such as allele–specific PCR [2, 6], minisequencing [7, 8], oligonucleotide ligation assay (OLA) [9, 10], ligase chain reaction (LCR) [11], and other complex methods such as the modified ligase chain reaction (Gap–LCR) [12], which is based on the combined use of both enzymes, have firmly established themselves in practical applications. For most of these methods, the oligonucleotide probes are designed in such a way as to place the putative substitution on the NA–template into the hybrid complex, thus forming a non–complementary base pair (mismatch). Therefore, detection of a mismatch coupled to the duplex–dependent labeling of the probe can take place at either of two stages: firstly, at the hybridization step (because of the lowered stability of the imperfect complex) and, secondly, at the probe’s enzymatic conversion step (because of the lowered effectiveness of the enzyme, caused by the presence of a mismatch in the DNA substrate) (Fig. 1). However, even such double control is not always sufficient for reliable DNA analysis. Single nucleotide mismatches which alter the complete complementarity of the DNA–duplex often do not provide sufficient specificity for a reliable diagnosis, even when DNA–dependent enzymes are used.

The search for enzyme–based ways to increase reliability in NA polymorphism analysis has been under way for several decades. But the issue is still unresolved, since the currently available methods for increasing the selective activity of DNA–dependent enzymes are often not universal and require preliminary screening in each specific case in order to optimize the procedure for the particular task.

This review summarizes data which characterize 1) The sensitivity of a range of DNA–dependent enzymes, which are used in NA analysis, to the presence and type of a single mismatch in the substrate complex; and 2) The structural traits of enzymes and substrate complexes which influence the selective activity of the enzyme. We analyze the peculiarities of the spatial organization of enzyme–substrate complexes, which encompasses a network of protein–nucleic junctions critical to the enzymatic reaction. We also review approaches to increasing the selectivity of enzyme–dependent reactions based on the introduction of additional “disruptive” elements into the structure of the imperfect DNA–substrate, such as artificial mismatches and synthetic nucleotide analogs.

## Selective activity of DNA-dependent enzymes towards non-complementary pairs in the structure of a DNA-substrate

This review uses the term “enzyme selectivity,” which is the ability of an enzyme to detect a non–complementary base pair in a substrate complex under certain conditions, thus lowering the effectiveness of the enzymatic conversion of the imperfect complexes as compared to perfect (fully complementary) ones. It is known that the ability of an enzyme to identify a certain non–complementary base pair in a DNA–substrate depends on the type of base pair, its nucleotide surroundings, and the location in regard to the site of the enzymatic conversion. The selective activity of enzymes also depends on several external factors, such as the buffering quality of the environment, temperature, and temporal conditions, so an analysis of the literature does not lead to an easy establishment of the general mechanisms of enzyme discrimination in some mismatches and tolerance towards others. Some of the difficulties in the analysis and comparison of the effective detection of mismatches are due to the different methods used for measuring the selectivity of enzymes in various studies. Most often, the authors compare the following characteristics: yield of the products of the enzymatic reaction, initial rates of product accumulation, and the ratio between V_max_ and K_m_. Usually, they consider the difference between the values of the threshold cycle (ΔС_t_) during a real–time PCR reaction for a perfect and imperfect template, or they analyze the occurrence frequencies of the mismatch in the products of the enzymatic conversion of a random oligonucleotide library paired into complexes with a DNA–template of known structure.

In general, a significant decrease in the amount of the resulting target product during elongation of a normal oligonucleotide probe is usually seen when the mismatch is on the exact 3’–terminus of the elongating chain [13–17], and it is sometimes also seen with the mismatch in the next–to–the–last position from the 3’–terminus [15, 18]. However, even several mismatches in the central part of the substrate complex have no noticeable effect on product accumulation. One study [19] showed that the presence of 2 to 4 inside mismatches in a long (28 ÷ 30 bp) DNA–oligonucleotide complex has no noticeable impact on the yield of the PCR reaction product (*Thermus aquaticus* DNA polymerase *(Taq)* was used in this reaction). Only the presence of 5 and 6 non–complementary base pairs lowered the efficiency of the PCR 22– and 100–fold, respectively. According to other data, a single nucleotide mismatch located farther from the 3’–terminus (up to the 8th position) is enough to lower the product yield of a PCR–reaction by a factor of 10 or more (up to 1,000) for probes 17– to 19–bp long [20]. It is notable that this was not observed on all the DNA–templates tested in this study [20].

The study of the type of mismatch on the 3’–terminus of the elongating chain, which affects the polymerase reaction, showed some general patterns (Table 1). Polymerases from different organisms show decreasing discrimination of nucleotide mismatches in the following order: Pur/**Pur** > Pyr/**Pyr** > Pur/**Pyr** = Pyr/**Pur** [2, 3, 14, 22, 23].

The normal font in the first position depicts the nucleotides from the oligonucleotide probe, while the second position bold text depicts bases from the template strand.

According to data from another study [3], the calculated relative elongation efficiency (V_max_/K_m_)_mismatch_/(V_max_/K_m_)_complement_ of a mismatch bearing DNA–substrate by *Taq* polymerase is less than 10^–6^ for a Pur/**Pur** , 10^–4^ to 10^–5^ for a Pyr/**Pyr** and 10^–3^ to 10^–4^ for Pur/**Pyr** and Pyr**/Pur** mismatches. Such measurements have also been performed for the * Drosophila melanogaster* DNA polymerase α and for the reverse transcriptase of the avian mieloblastosis virus (AMV RT) [23]. Discrimination efficiency for each mismatch type is about 10 times less for these enzymes. In general, accumulation of a mismatch–bearing product is in complete agreement with the presented scenario, but complete inhibition of the enzyme and absence of the full–size product can only, if rarely, be seen in the case of purine/purine mismatches [2, 3]. However, some exceptions to this general rule have been reported. Studies [3, 14] show that *Taq* DNA–polymerase does not elongate a primer if it has a pyrimidine/pyrimidine С / **С** mismatch at its 3’–terminus; however, it does efficiently elongate the primer if there is a С / **T** mismatch in the primer–template complex at the same position [3], or for that matter any other mismatch with a Т residue on the 3’–terminus of the primer (T/**G**, T/**C**, T/**T** ) [14]. According to another study [15], primers that have T, G or C on the 3’–terminus do not get elongated by * Taq* polymerase if the template bears substitutions in this position, while primers with 3’–A show low discrimination of any of the 3’– А / ** N** mismatches, albeit with a decrease in the effectiveness of the whole PCR reaction.

**Table 1:** Relative elongation effectiveness for 3’-terminal mismatch bearing DNA-complexes; reaction catalyzed by DNA-polymerases

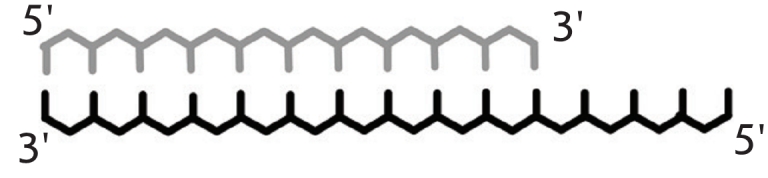 Perfect complex	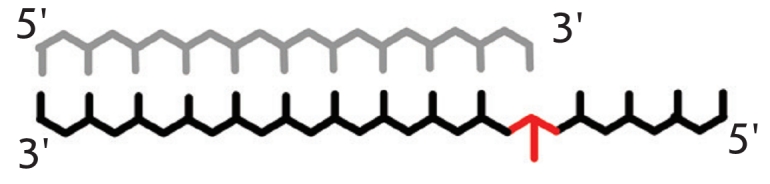 Mismatched complex

DNA-polymerase	Respective template nucleotide	primer, 3'-terminal nucleotide				reference
A	T	G	C
*Taq* DNA-polymerase	T	++++	+	++	+	[15]
	A	+	++++	+	+	
	C	+	+	++++	+	
	G	+	+	+	++++	
*Taq* DNA-polymerase	T	++++	++++	++++	++++	[14]
	A	++	++++	+	++++	
	C	++++	++++	++++	+	
	G	+	++++	+++	++++	
*Taq* DNA-polymerase	T	++++	+++	++++	+++	[21]
	A	++	++++	+++	+++	
	C	++++	+++	++++	+++	
	G	++	+++	+++	++++	
*Taq* DNA-polymerase	T	++++	+	++	+++	[3]
	A	+	++++	–	++	
	C	++	+	++++	–	
	G	–	++	–	++++	
Avian myeloblastosis virus reverse transcriptase	T	++++	++	++++	++	[23]
	A	+	++++	+	++	
	C	++	++	++++	+	
	G	+	+++	–	++++	
*Drosophila melanogaster* DNA-polymerase α	T	++++	++	++	++	[23]
	A	+	++++	+	++	
	C	++	++	++++	+	
	G	++	++	+	++++	

Comment. «–» means that there was no observable product of the conversion of the oligonucleotide probe. When the product does appear, the effectiveness of this process is rated by a four-step scale from «+» to «++++». The effectiveness of product formation from a complementary template was always taken as «++++».

As for DNA–polymerases, the position of an oligonucleotide mismatch in relation to the conversion site is a major factor in determining the effectiveness of an enzymatic reaction catalyzed by DNA–ligase. Close proximity of a mismatch to the ligation site between two oligonucleotides (single–strand break) increases the mismatch discrimination factor, causing very effective enzyme inhibition [13, 24, 25]. For instance, T4 phage and *Thermus* *thermophilus (Tth)* bacterial DNA–ligases show reaction yields 2.5 to 5 fold lower when the mismatch in the substrate is located one nucleotide off the conversion site, as compared to when it is squarely in the site [24]. Ligation of random sets of nanomers [26] and dodecamers [27] onto a DNA–template using *Tth* DNA ligase and *E.coli* DNA–ligase, respectively, showed, after sequencing of the long products, that the largest number of mismatched base pairs were located in the central parts of the complexes of ligated oligonucleotide blocks. Mismatches were primarily situated in the 5^th^ position from the 3’–terminus in case of nanonucleotides [26] and in the 6^th^ of 7^th^ position from the 3’–terminus in case of dodecanucleotides [27]. Ligation of probes bearing random nucleotides in the first 5 positions from each side of the single strand break showed that mismatches were rarely found in the first two positions on either side of the nick in case of the Т 4 phage DNA–ligase. The largest number of mismatches was seen in the 3^rd^ position, and the mismatch most often occurring was T/**G** [28]. It is worth noting that besides the proximity of a mismatch to the single–strand break, DNA–ligase discrimination is also affected by the component of the ligation in which the mismatch is situated: in the duplex part of the 3’–hydroxyl donor ( ОН –component) or in the 5’–terminal phosphate donor (P–component). DNA–ligases impose “extra” requirements on the structure of the ОН –member of the substrate complex, and any disruption in this part of the DNA–complex has a much more pronounced effect on the enzymatic process than a similar disruption in the Р –component of the duplex [26–32].

It is difficult to establish a general rule for the effects of mismatches of different types located in close proximity to the conversion site on DNA–ligases from various organisms (Table 2) based on the analysis of data in the literature. *Vaccinia virus* and *Chlorella virus* ligases, as well as human DNA–ligases I and III, can effectively discriminate only 3’–purine/purine mismatches located in the ОН –component [29, 30, 33, 34]. The *Chlorella virus * DNA–ligase also exhibits a significant (100–fold) decrease in ligation efficiency, as compared to perfect substrates, in case of 5’–G/**A** and A/**G** mismatches in the P–component [29]. Most oligonucleotide mismatches in the Р –component of the complex practically cannot be discriminated by the above–mentioned DNA–ligases. Thymidine–bearing mismatches 5’– С / ** T ** and G/ ** T ** are the worst discriminated ones [33]. The archeal * Thermococcus kodakaraensis* DNA–ligase is sensitive to any 3’–mismatch of the ОН –component and can only discriminate 5’–terminal Р –component mismatches if they are purine/purine [31]. DNA–ligase from the African swine fever virus (ASF) is one of the least sensitive to 3’–mismatches in the OH–component when compared with the other DNA–ligases studied [35]. This DNA–ligase shows the highest fidelity (calculated according to (V_max_/K_m_)_mismatch_/(V_max_/K_m_)_complement_, which is 10^–4^ towards a substrate with a 3’–G/ ** A** mismatch ** , ** and the lowest (2.7) towards a complex with a 3’– Т / ** С ** mismatch, which is converted more effectively than perfect substrates. In case of the *Tth* DNA–ligase, ligation of random selection of probes showed that the prevalent mismatches were those containing purine (G/ **T ** , G/ ** A ** , G/ ** G ** , A/ ** G ** ), which amounted to 86%, and 71% of the non–complementary pairs bore a guanine residue [26]. Also, a nonequivalence of isomismatches (G/ ** T** and T/ ** G) ** was noted during library ligation. The occurrence frequencies were 14 and 2 times (the overall number of mismatches was 57) for guanines located in the ligated and the template strands, respectively [26]. A similar oligonucleotide library ligation experiment with * E.coli* DNA–ligase showed prevalent accumulation of G/ ** T ** mismatches [27]. Also, several studies demonstrated that T4 DNA–ligase can ligate most mismatches irrespective of their location relative to the site of enzymatic conversion [32, 36, 37]. Nevertheless, [35] shows that 3’–terminal purine/purine mismatches, with the exception of 3’–G/ ** G ** and the pyrimidine/pyrimidine mismatch 3’– С / ** С  ,** can be discriminated, since the fidelity of their conversion by Т 4 DNA–ligase is approximately 10^–4^ to 10^–6^, as calculated using the above–mentioned formula.

**Table 2:** Relative effectiveness of the ligation of DNA-complexes, which are either perfect, or bear a mismatch on the 3’-terminus of the OH-component or on the 5’-terminus of the Р-component; catalysis by DNA-ligases

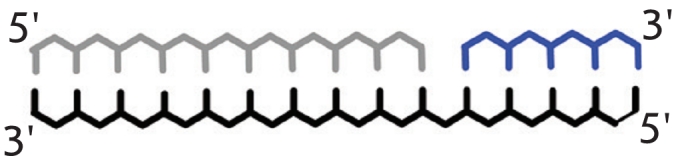 Perfect complex	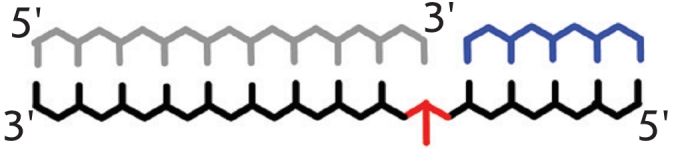 Complex with a 3’-terminal mis-match of the OH-component	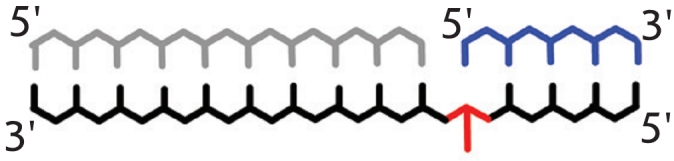 Complex with a 5’-terminal mis-match of the P-component

DNA-ligase	Respective template nucleotide	ОН-component, 3'-nucleotide				Р-component, 5'-nucleotide				reference
A	T	G	C	A	T	G	C
Т4 DNA-ligase	T	++++	++++	++++	+++	++++	++++	++++	++++	[36]
	A	++++	++++	+++	++++	++++	++++	++++	++++	
	C	++++	++++	++++	+++	++++	++++	++++	+++	
	G	++++	++++	++++	++++	++++	++++	++++	++++	
Т4 DNA-ligase	T	n/a	n/a	n/a	n/a	++++	+++	++++	+++	[37]
	A	n/a	n/a	n/a	n/a	++	++++	+	+++	
	C	n/a	n/a	n/a	n/a	++++	+++	++++	++	
	G	n/a	n/a	n/a	n/a	+	++++	+	++++	
Т4 DNA-ligase	T	++++	+++	++	+++	n/a	n/a	n/a	n/a	[35]
	A	++	++++	+	+++	n/a	n/a	n/a	n/a	
	C	+++	++++	++++	++	n/a	n/a	n/a	n/a	
	G	++	++++	+++	++++	n/a	n/a	n/a	n/a	
Human DNA-ligase III	T	++++	+	+	++	n/a	n/a	n/a	n/a	[33]
	A	+	++++	–	+	n/a	n/a	n/a	n/a	
	C	+	++	++++	+	n/a	n/a	n/a	n/a	
	G	–	+++	–	++++	n/a	n/a	n/a	n/a	
*Chlorella virus* DNA-ligase	T	++++	+	+	++	++++	++++	++++	++++	[29]
	A	++	++++	+	++	++	++++	+	+++	
	C	+	++	++++	+	+++	+++	++++	++	
	G	+	++	+	++++	+	+++	++	++++	
*Tth* DNA-ligase	T	++++	–	+	–	++++	++	++	–	[30]
	A	–	++++	–	–	+	++++	–	+	
	C	–	–	++++	–	++	+	++++	–	
	G	–	+	–	++++	+	+	–	++++	
ASF virus DNA-ligase	T	++++	+++	+++	++++	n/a	n/a	n/a	n/a	[35]
	A	++	++++	++	++++	n/a	n/a	n/a	n/a	
	C	+++	++++	++++	+++	n/a	n/a	n/a	n/a	
	G	++	+++	+++	++++	n/a	n/a	n/a	n/a	

Comment. See comment to Table 1; n/a – effectiveness of product formation was not assessed.

Literature sources on the identification of single mismatches in proximity to the enzymatic conversion site of DNA–polymerases and DNA–ligases are listed in Tables 1 and 2, respectively. The data presented confirm all of the discussed peculiarities of the reactions catalyzed by DNA–dependent enzymes on DNA–substrates with a single mismatch.

Thus, the only fully confirmed fact is that DNA–polymerases and DNA–ligases do not always exhibit absolute selective activity when converting natural duplexes, which would be needed for the reliable detection of any nucleotide mismatches in a substrate complex formed from native oligonucleotides. This means that one of the foremost goals in the development of approaches to detect point mutations using DNA–dependent enzymes is a systematic analysis of the factors that influence the sensitivity of these molecular systems to local disruptions in the probe–DNA complexes. In our view, specific data on the structure of the diagnostically useful enzymes, DNA–ligases and DNA–polymerases, as well as studies of the spatial structure of substrate complexes, will make it easier to explore possible ways to increase selectivity in DNA analysis and to gauge the effectiveness of such ways.

The specifics of the spatial structure of DNA–polymerases, DNA–ligases and their complexes with DNA–substrates

DNA–polymerases and DNA–ligases catalyze the formation of new phosphodiester bonds between the nucleotide precursor–components that make up two–strand DNA structures. Even though these are two separate classes of enzymes, they are similar in many ways, such as common structural elements and similarities in the interaction with the DNA–substrate.

## Common characteristics of the domain organization and active site structure of DNA-dependent enzymes

**Fig. 2:**
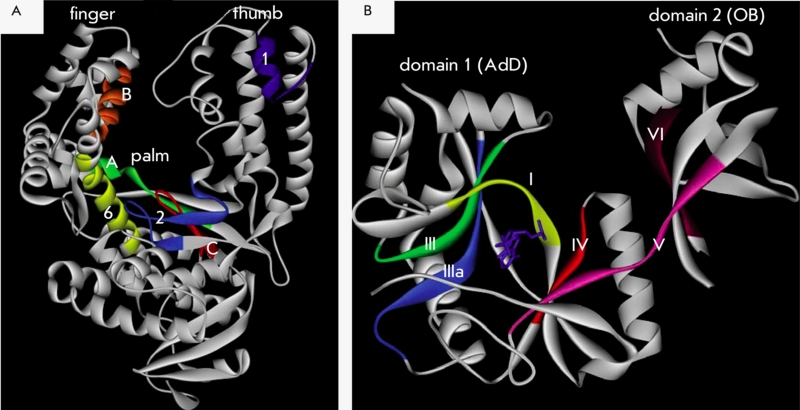
Spatial and domain structure of Taq DNA-polymerase in its free state (А) and Chlorella virus DNA-ligase in adenylated state (B). Distinct motifs are highlighted with colors. Images were obtained using PDB 1TAQ [41] and 1FVI [54], respectively

The catalytic cores of DNA–polymerases extracted from different organisms have varying amino–acid sequences and belong to different families, but they still have a similar structure and consist of three domains, which are assembled in a structure reminiscent in shape of a half–open palm. The domains have appropriate names such as “palm,” “thumb,” and other “fingers” (Fig. 2, А) [38–40]. The domains of the A–family DNA–polymerases consist of six evolutionarily conserved motifs ( А , В , С , 1, 2 and 6), which are thought to play the main role in the formation of the active site and the network of specific bonds with the DNA–substrate [39–42]. The most conserved motifs are А , В and С , two of which ( А and С ) are present in all the known DNA– and RNA–polymerases. Motifs 1, 2 and 6 also have a fairly conservative spatial structure, but they show more variety in terms of amino acid sequence. Compared to the highly conservative А , В and С domains, these other domains are less involved in forming bonds with DNA. To capture the dsDNA–substrate, the enzyme uses the “palm” (motifs А , 2, 6) and the “thumb” (motif 1) domains. The “fingers” domain closes above the “palm” forming a pocket (cavity) for the newly formed base pair. This pocket is mainly made up of motif B amino acid residues. The fragments responsible for the capture of the 3’–terminus of the primer, the inserted nucleotide, and the two magnesium ions needed for the catalysis are all localized on the inner surface of the “fingers” (motifs В , 6) and on the surface of the “palm” at the base of the “fingers” (motifs А , С ). The polymerase active site, which accomplishes the addition of nucleotides to the growing strand, is situated in the “palm” domain [40, 42]. Some DNA–polymerases also have additional domains, which can, for instance, add 3’ → 5’ exonuclease activity.

**Fig. 3:**
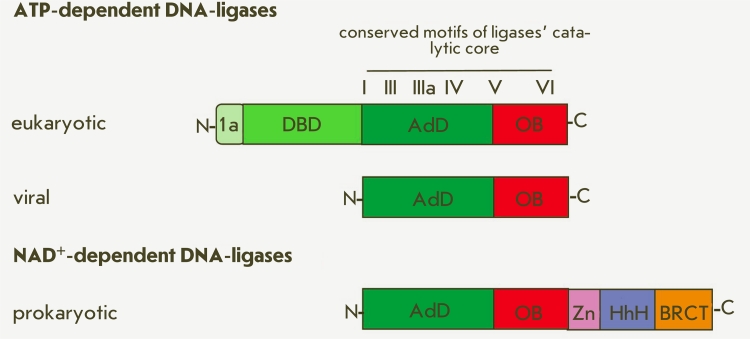
Domain structure of DNA-ligases.

Like DNA–polymerases, DNA–ligases extracted from various organisms have a common minimal catalytic site, which is formed by two distinct domains: the N–terminal (AdD) catalytic domain and the smaller С –terminal ( ОВ ) regulatory domain (Fig. 2, B). The ligase active site is for the most part made up of six conserved motifs (I–VI). Five of them (I, III, IIIa, IV and V) are parts of the N–terminal domain 1 (AdD) and form the nucleotide–binding cavity. This domain is responsible for the identification and specific bonding with ATP (or NAD^+^), and, there, motif I contains a lysine residue which is adenylated during the enzyme’s activation. Through motif V, domain 1 binds to the oligonucleotide/oligosaccharide binding domain 2 ( ОВ ) [43–46]. Binding of the DNA–substrate to the enzyme takes place through the interdomain crevice including several structures, such as motif V [43]. Besides the regular domains, eukaryotic ligases carry an additional DNA–binding domain (DBD) on their N–terminus, which is vital for catalysis and allows a tighter “grip” on the DNA duplex [44, 47]. The C–terminus of NAD^+^–dependent ligases carries the “zinc fingers” (Zn), “helix–hairpin–helix” (HhH), and other domains. These are analogous to the DBD–domain, and they increase the efficiency of substrate binding and/or the fidelity in discriminating disruptions in the substrate structure [44] (Fig. 3).

The structural organization of DNA–ligases, just like that of DNA–polymerases, is comparable to a hand. Domain 1 is called the palm; and domain 2, the fingers [48]. Such a metaphoric view of these domains of DNA–dependent enzymes is useful when analyzing the structural rearrangements they undergo during enzymatic reactions.

## Conformational changes during enzymatic reaction

**Fig. 4:**
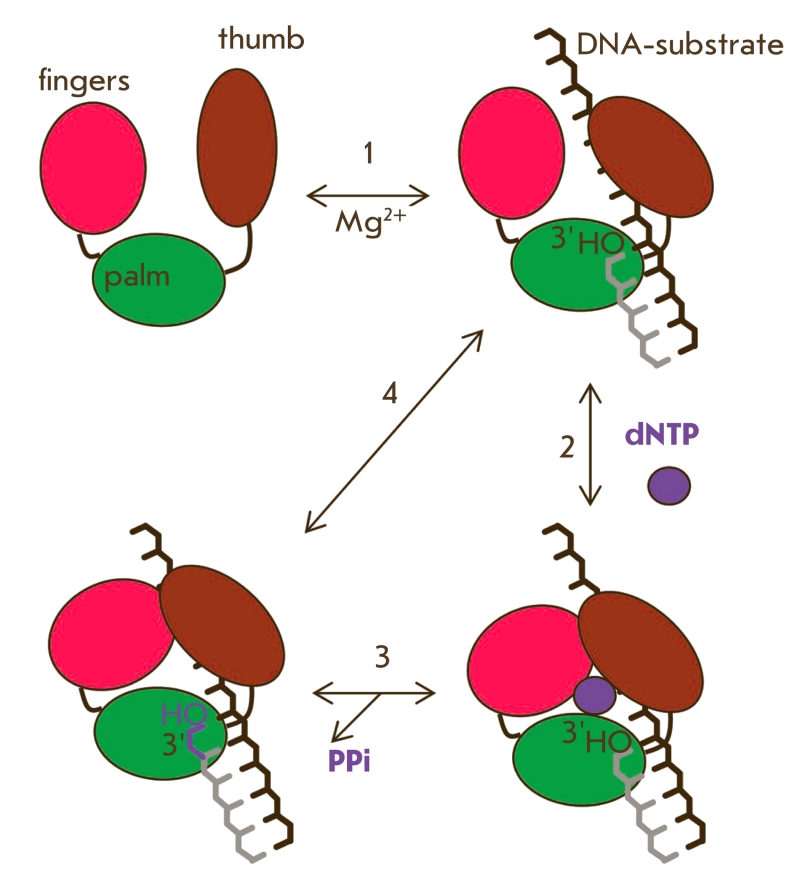
Schematic representation of the conformational changes in DNA-polymerases during the enzymatic elongation of a DNA-substrate.

In order to perform effective catalysis, the molecules of DNA–processing enzymes undergo conformational transitions. During a catalytic cycle, polymerases experience two main structural changes (Fig. 4). The first is coupled with the binding of the DNA–substrate, which enters the open crevice between the “thumb” and the “palm” of the enzyme. The upper edge of the “thumb” interacts with the substrate from the side of the minor groove of the double helix, and thus it bends towards the surface of the palm. This causes the “thumb” domain to form a hollow cylinder, which has a fragment of the DNA–helix firmly lodged inside. Then, the second conformational change occurs; the “fingers” turn towards the “palm,” which is coupled with the binding of a nucleosidetriphosphate molecule in the polymerase’s active site. This change is called the transition between the “open” and “closed” states of the enzyme, and it is the final positioning and binding of the substrate in the enzyme’s active site. This is the step when the bonds between the “fingers” domain and the inserted nucleotide form, which allows to analyze the geometry of the transitional state, and thus the complementarity of the forming base pair [49–51].

**Fig. 5:**
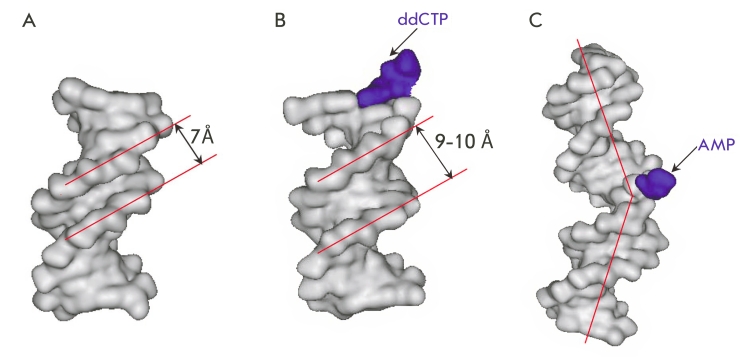
Structure of a DNA-duplex in B-form (PDB 1BNA 12 bp) [53] (А), a hybrid form of DNA-duplex in the Taq DNA-polymerase active site (PDB 3KTQ 12–13 bp) [51] (B), and Chlorella virus DNA-ligase (PDB 2Q2T 21 bp) [57] (C).

Changes in the enzyme’s structure are accompanied by adjustments in the DNA–substrates, which mainly take place at the stage where the duplex/polymerase complex is assembled. The vicinity of the DNA–duplex close to the enzymatic conversion site experiences changes of the carbohydrate–phosphate backbone: namely, the transition of deoxyribose residues from the С 2’–endo to the С 3’–endo conformation. This causes significant changes in the shape of the minor groove; it becomes wider and shallower. The width of the groove increases significantly from 7 Å (DNA–helix in a free state) to 9–10 Å (in a complex with an enzyme). Thus, a fragment of the DNA–duplex helix is transformed from the B–form into an A–like form [49, 51, 52] (Fig. 5). These structural changes in the enzyme–bound DNA–substrate involve no more than 4–5 bp [49, 51, 52]. When deoxynucleosidetriphosphate is bound and the complex becomes “closed,” the substrate experiences additional conformational changes that involve the single–strand piece of the template chain, which appears to be fixed [51].

**Fig. 6:**
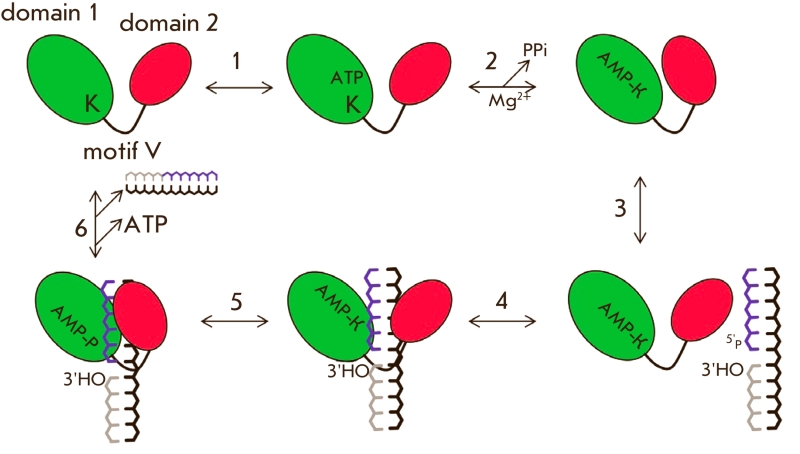
Schematic representation of the conformational changes in DNA-ligases during the enzymatic ligation of a DNA-substrate with a single-strand break. К – conserved lysine residue, which is adenylated at the first step of the enzymatic reaction.

DNA–ligases also experience a conformational transition from “open” to “closed” when performing their catalytic activity. “Closing” of the enzyme begins after the nucleotide cofactors АТР or NAD^+^ have reacted, which causes the mobile domain 2 to come into close proximity with domain 1 (Fig. 6). An АТР (NAD^+^) molecule is coordinated in a position favorable for a nucleophilic attack of the ε –aminogroup of the conserved lysine residue at the α –phosphate of АТР (NAD^+^). Moreover, such a structural change leads to the formation of the catalytically active site and turns the DNA–binding domain towards the active site [44, 47, 53, 55]. Such changes not only cause the adenylated enzyme to take on a conformation which can recognize and capture DNA–substrates [44]. Final “closing” of the enzyme happens upon its binding to DNA and leads to the tight surrounding of the DNA–substrate by the adenylating and ОВ domains, as well as by the DBD or HhH domains, in the vicinity of the single–strand break.

Similarly to DNA–polymerase data, an opinion has been voiced to the effect that the DNA–substrate which is in complex with DNA–ligases switches from B– to A–form [48]. One of the proven facts bolstering this hypothesis is that the cavity between the DNA–ligase domains, where the binding of the duplex happens, is crooked, and in fact the warped B–A hybrid DNA–duplex would structurally match this site of the active enzyme [47]. These changes in the substrate have been confirmed by X–ray structural studies on DNA–ligase I complexes with human DNA [47]. In this case, deformation of the DNA helix into an A–like shape was only seen in part of the duplex on the ОН –component side. Similar data obtained for DNA–ligases from the * E.coli * and * Chlorella * viruses [56, 57] also suggest that, upon binding to DNA, the regions situated on both sides of the nick partially revert to the A–form (Fig. 5, В). The length of such partially unwound DNA fragments is dependent on the specific enzyme and varies from one to six nucleotide pairs [47, 48, 55–57].

Notably, the В → А transition of the DNA helix has been reported for other DNA–dependent enzymes (such as DNAs e I ), as well as for DNA–binding proteins [58]. The main reason for such a transformation of the substrate is thought to be rapid dehydration of the double helix in the hydrophobic DNA–binding cavity of the enzyme, which promotes this change in the dsDNA molecule.

## Protein-nucleic interactions in the reactive enzyme/DNA-substrate complex

Changes in the structures of the enzyme and the substrate “tune” both of them to each other, creating a whole network of protein–nucleic acid interactions based on hydrogen and ionic bonds, as well as on Van–der–Waals interactions. This network of bonds is highly specific, and the residues of the active site, which are the most conservative ones, are often incorporated into this network. Unwinding of the DNA–duplex near the enzyme’s active site increases the availability of various sites in the minor groove of the double helix, which can in turn interact with the protein structure. For the most part, these interactions are tight nonsequence–specific interactions, which are based on hydrogen bonds between the centers present in any canonic base pair (electron acceptors, which are in the N3 position of purine residues and in the O2 position of pyrimidine residues) and the conserved amino acids in the protein [51, 52, 59, 60]. In turn, the induced A–form of the duplex is stabilized by a network of Van–der–Waals interactions between the amino acid residues and the carbohydrate fragments and/or heterocyclic bases of the nucleotide [43, 47, 51, 52].

**Fig. 7:**
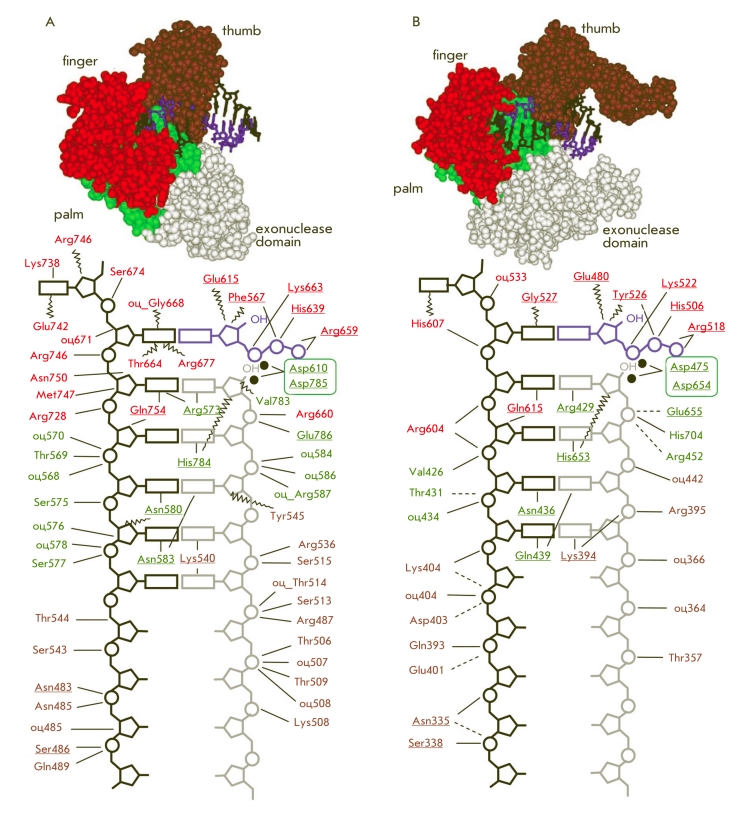
Schematic representation of the network of protein-nucleic interactions, which are formed between the DNA-substrate, dNTP and Taq DNA- polymerase [51, 61] (A) or Т7 DNA-polymerase [49] (B). Direct contact is depicted as solid lines, water-molecule mediated contact is depicted as dotted lines, Van-der-Waals interactions as wavy lines. Amino acids belonging to different domains of the enzyme are depicted in different colors. Highly conservative residues in the A-family polymerases are underlined. Interactions formed by the atoms of the main chain of the polypeptide have the “mc” prefix. Metal ions are depicted as black circles. The upper panel depicts the spatial structures of the respective enzyme-substrate complexes obtained using PDB structures, 3KTQ [51] (А) and 1T7P [49] (B).

The region of the DNA that interacts with a polymerase via the minor groove of the duplex structure is about 4–5 base pairs long and is located on the 3’–terminus of the primer strand. X–ray diffraction analysis reveals which amino acid residues in the A–family polymerases ( * Taq, Bst, * etc.) are involved in the formation of bonds in the groove (in case of the * Taq * DNA–polymerase):

- arginine, Arg573, which forms a hydrogen bond both with the 3’–terminal nucleotide of the primer and its complementary nucleotide in the template;
- glutamine, Gln754, which interacts with the same template nucleotide;
- asparagine (Asn583) and lysine (Lys540), which form bonds with the 3^rd^ , and 4^th^ and 5^th^ 3’–terminal nucleotides of the elongating strand, respectively [51, 61, 62] (Fig. 7).

Thus, the length of the DNA–complex in which the minor groove is involved in the formation of hydrogen bonds is the same as the DNA–fragment that converts to the A–form. Notably, the nucleotides which are near the enzymatic conversion site not only take part in the bonds in the minor groove, but also participate in Van–der–Waals interactions with several amino acid residues (histidine, arginine, tyrosine), which promote the fixation of the DNA–strand in its A–form [43, 47, 51]. The DNA outside this region, i.e. farther than 4–5 bp from the elongation site, is virtually all in the B–form, which makes protein–nucleic interactions possible only through the carbohydrate–phosphate backbone, making these interactions nonsequence–specific [43, 51, 52]. Interactions in this region of the enzyme–substrate complex are mainly of electrostatic and Van–der–Waals nature. A–family polymerases form various contacts with 5–8 3’–terminal base pairs of the DNA duplex [43, 51, 52, 61], and more than 40 conserved amino acids take part in these interactions [63–65] (Fig. 7, А, B).

The crystal structures of the DNA–ligase/dsDNA–substrate complex, which imitates the reactive state, were obtained and characterized only recently and only for a few enzymes. Before that, researchers knew only the lengths of the DNA–ligase binding sites on the substrate, which were identified by various foot–printing methods. Studies showed that the sizes of the regions which are covered by the enzyme on both sides of the single strand break are uneven. The enzyme binds 7–12 bp on the P–component side and 3–9 bp on the ОН –component side [66, 67]. The overall interactive region of the DNA–substrate with the ligase varies from 10 to 20 bp depending on the enzyme. For instance, the ATP–dependant T7 bacteriophage DNA–ligase (41 kDa, 359 amino acids (aa)) was shown to bind * Taq, Bst * , etc., 3–5 bp in the 3’ → 5’ direction from the nick and 7–9 bp in the opposite direction along the ligated strand, using foot–printing methods [66]. Enzymatic (exonuclease III) foot–printing showed that the * Chlorella virus * ligase (34 kDa, 298 а a) can cover 19–21 bp of the DNA–substrate, of which 11–12 bp are on the 5 ′ –phosphate donor side, and 8–9 bp are on the other side of the nick [67]. Studies [13, 68] show that Т 4 DNA–ligase (55 kDa, 487 а a) exhibits 11–12 bp and 6–7 bp and 5 bp regions, respectively (values are presented as in the previous example).

**Fig. 8:**
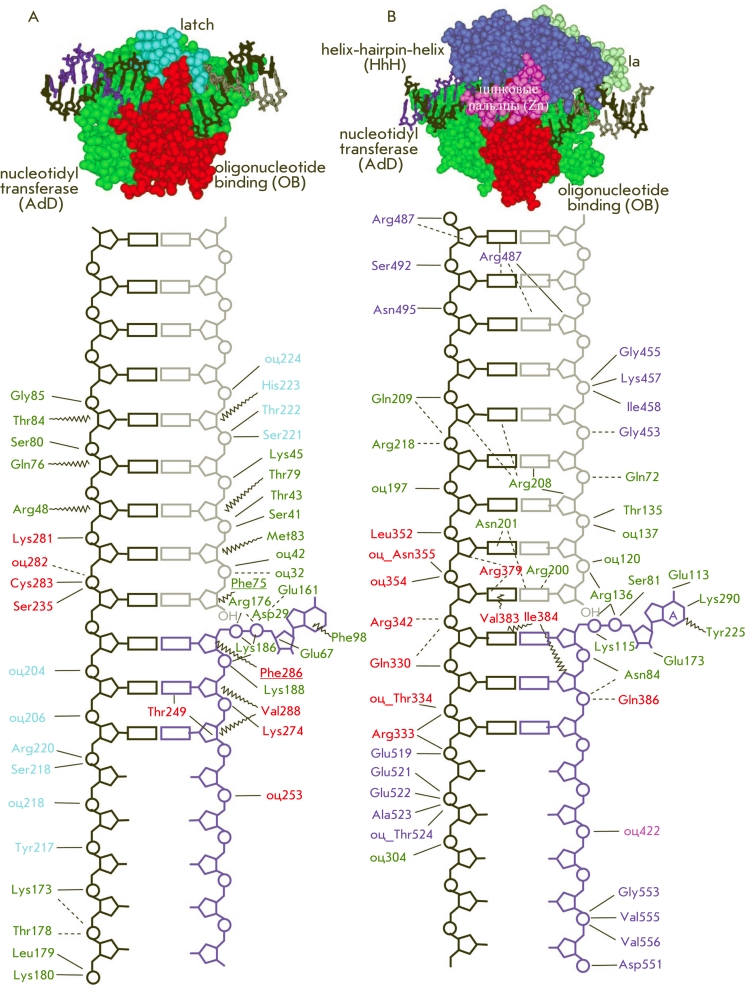
Schematic representation of the network of protein-nucleic interactions, which are formed in the reactive complex between the adenylated DNA-substrate and the Chlorella virus DNA-ligase [57] (A) or the E.coli DNA-ligase [56, 69] (B). Direct contact is depicted as solid lines, watermolecule mediated contact is depicted as dotted lines, Van-der-Waals interactions in wavy lines. Amino acids belonging to different domains of the enzyme are depicted in different colors. Interactions formed by the main chain of the polypeptide have the “mc” prefix. The upper panel depicts the spatial structures of the respective enzyme-substrate complexes, obtained using PDB structures 1FVI [51] (А) and 2OWO [56] (B).

Currently, there is an opportunity to systematize the interactions of DNA–ligases with substrates and to determine which amino acid residues are similar in functions in enzymes extracted from various sources. It is known that the ligase/DNA–substrate complex is formed by a network of bonds which coordinate the 5’–terminal phosphate residue of the P–component, involve the minor groove of the DNA–duplex near the single–strand break, as well as the carbohydrate–phosphate backbone of each of the DNA–substrate’s strands [47, 56, 57, 69, 70]. Figures 8, А and B show a map of the tight interactions which take place between the substrate and the DNA–ligases of ** the * Chlorella * virus and *E.coli * . It was proved that the bonds with the DNA–substrate involve all the domains of the enzyme. The major parts of these point intermolecular interactions are hydrogen bonds, which occur between the amino acid residues and the phosphate moieties of the DNA. X–ray diffraction data indicate that these interactions involve approximately 8 to 5–6 bp from the Р – and ОН –components of the substrate, respectively [56, 57]. Bonds between the heterocyclic base moieties, which are exposed into the minor groove, and the amino acid residues of the enzyme involve only two nucleotide pairs on each side of the single–strand break [56, 57]. That is the precise length of the helical region of the duplex that experiences a В → А transition during the formation of a complex with the enzyme [57]. The same region of the DNA–substrate is involved in the formation of Van–der–Waals interactions with ligases. For a whole range of DNA–ligases, these bonds are formed due to the intercalation of arginine and/or phenylalanine residues between the carbohydrate residues exposed in the minor groove of the DNA–duplex [56, 57]. In the case of eukaryotic and NAD^ + ^–dependent enzymes, DBD and HhH domains play a very significant role in the formation of bonds in the minor groove [56, 57, 69, 70]. Moreover, a bend in the DNA–substrate in the enzyme’s active site has been demonstrated for a number of DNA–ligases (Fig. 5, C). In the case of the *E.coli * DNA–ligase, it has been shown that the HhH domain forms bonds with the phosphodiester backbone at four positions, thus stabilizing the bend of the main DNA–axis (~10°) near the nick [57] (Fig. 8, B). The authors note that the HhH motif, which is formed by five α –helical sub–motifs, has been found in many DNA–binding proteins [57, 71, 72].

## Factors influencing the effective processing of the DNA-substrate during the enzymatic reaction

There are many hypotheses on the factors affecting the sensitivity of DNA–dependent enzymes towards noncanonic base pairs in the part of the DNA–substrate that is recognized by the enzyme. These factors determine the selective activity of the enzymes including DNA–ligases and DNA–polymerases. Several of these factors will be discussed further.

The presence of canonic Watson–Crick hydrogen bonds near the processed region of the substrate and/or their stability, as well as the overall stability of the substrate complex, was long thought to be the criteria determining enzymatic catalysis in model systems based on oligonucleotides. The mechanism which helps achieve the selective conversion of substrates is still not fully understood. More and more facts indicate that the contacts between the enzyme and the substrate not only bind and adjust the latter in the active site, but also help identify substrates with mismatches or any other disruptions of the regular DNA structure.

**Fig. 9:**
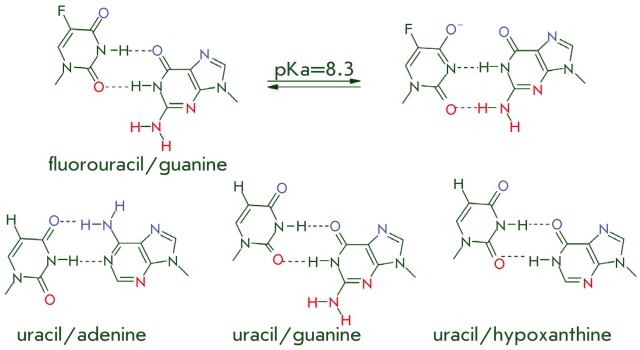
Modified non-canonical base pairs. Blue and red colors depict atoms which are donors/acceptors of hydrogen bonds and face the major and minor groove, respectively.

One of the criteria which allow enzymes to identify mismatches in the DNA may be the disrupted structure of the DNA helix caused by a mismatched base pair. This hypothesis is confirmed by experiments with 5–fluorouracil, which forms a pair with guanine (Fig. 9). Such a modified pair is “swinging,” which means that depending on the рН of the solution it can be in a paired state, similar to the Watson–Crick С /G pair, or assume a different state (Fig. 9) [73]. The presence of such a stable, but structurally noncanonic base pair in the recognition site considerably lowers the reaction yield for *Tth* and Т 4 DNA–ligases as compared to the 5–fluorouracil/guanine pair, which is similar in geometry to a normal complementary pair [73]. The noncanonic nucleotide pairs uracil/guanine and uracil/hypoxanthine (Fig. 9) also lower the effectiveness of ligation of DNA–complexes, which have such pairs in the single–strand break site, by *Tth* ligase. It has been demonstrated that slowing of this enzymatic reaction takes place at the stage of DNA–substrate adenylation [73].

**Fig. 10:**
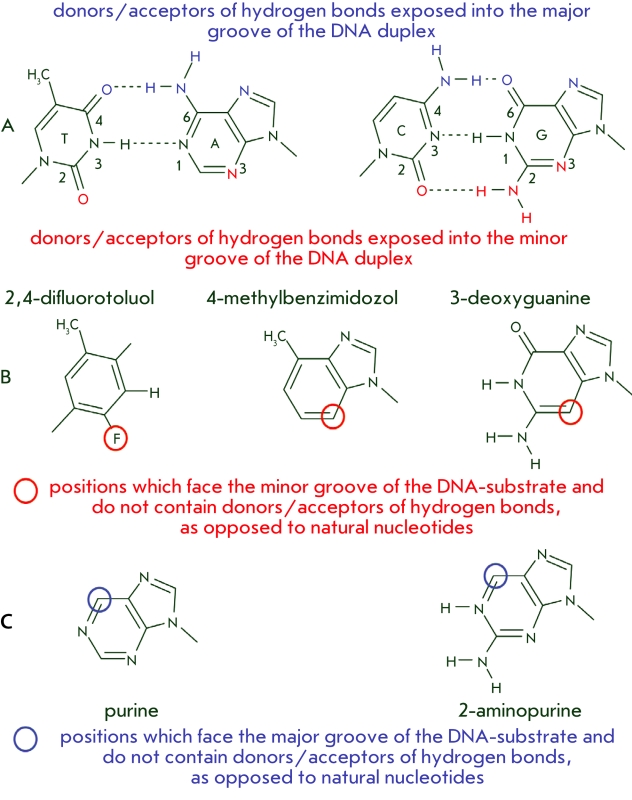
Layout of the hydrogen bond donors/acceptors in the minor and major grooves for the Watson-Crick base pairs (А). Modified bases, which do not have hydrogen bond donors/acceptors in the appropriate positions in the minor (B) or major (C) grooves. Blue and red colors depict the atoms which are donors/acceptors of hydrogen bonds and face the major and minor groove, respectively.

Another aspect of the selectivity mechanisms of the enzymatic process is the formation of hydrogen bonds between the enzyme and the minor groove of the substrate DNA. It so happens that the hydrogen bond acceptors of heterocyclic bases, which face onto the minor groove, all have a typical layout in case of correct pairing (bearing in mind the different roles of the processed and template strands of DNA–substrate) and an atypical one for noncomplementary pairs [59] (Fig. 10, А). Such a topological trait can promote the identification of mismatches and perfect pairs. The importance of such bonds is indirectly confirmed by the fact that human β DNA–polymerase and HIV–1 RT (reverse transcriptase) form only a single bond in the minor groove of the DNA–helix and are characterized by indiscriminate elongation of DNA–duplexes with mismatches, as opposed to A–family polymerases, which form multiple contacts [60, 74].

The role of enzyme–substrate bonds in the minor groove was studied using both nucleotide analogs in the DNA and mutant enzymes. It is clear from the structures of the enzyme–substrate complexes presented earlier that the base pairs in the enzymatic conversion site are always involved in hydrogen bonds with amino acid residues. In order to asses the importance of these contacts, a single nucleotide in this pair is replaced by a nonnatural analogue, such as 3–deazoguanine (guanine analog) [75, 76], 2,4–difluorotoluol (thymidine analog) [73, 77], or 4–methylbenzimidazol (adenosine analog) [77] (Fig. 10, B). The presence of such modified nucleotides, which are similar to their natural analogs, but do not have hydrogen bond acceptors in the minor groove, is one of the causes behind the termination of the enzymatic reaction catalyzed both by DNA–polymerases (Klenow fragment [75, 77], * Taq * , Т 7, HIV RT, polymerases α and β [77]) and DNA–ligases ( *Tth* and Т 4) [73]. The presence of the modified analog significantly lowers the effectiveness of DNA–polymerase catalysis only when the analog is present in the enzymatic conversion site [77]. Introduction of the analog nucleotides into both strands of the substrate shows that for the tested DNA–polymerases hydrogen bond formation in the minor groove is required only on the primer strand side for most polymerases, and that it is needed in the template component only for HIV RT [77].

Investigations of the role of minor groove interactions from the point of view of the enzyme’s structure were conducted using mutant forms of the Klenow fragment of the *E.coli * DNA–polymerase I [76, 78, 79]. Mutants R668A and Q849A have amino acids involved in the formation of hydrogen bonds in the wild–type enzyme substituted. These two amino acids form bonds with the heterocyclic bases of the 3’–terminal nucleotide pair of the elongated and template strands, respectively. These functional amino acid residues are replaced by alanine, which does not have hydrogen bond donors in its side chain. The R668A substitution causes reduced effectiveness of the enzyme–substrate interaction with a perfect complex and has a very little altering impact on the processing of a 3’–mismatched substrate [76, 78, 79]. The Q849A substitution does not affect the identification of the DNA–substrate by the enzyme [78]. Thus, the study of mutant enzyme forms of DNA–polymerases confirms the importance of bonds in the minor groove as a factor that determines effective processing of the substrate. In this case, as well as in the case of use of modified nucleotides, the formation of hydrogen bonds in the minor groove of the DNA molecule was most important in the elongated strand.

There is data that suggest that the effectiveness of the enzymatic reaction does not depend on the nature of the side–chains facing the major groove of the substrate DNA, nor does it depend on the moieties at the 6^th^ position of the heterocyclic base. Two such nucleotide analogs are used; 2–aminopurine and purine. Both of them can form a pair with uracil (Fig. 10, C) [73]. The presence of modified bases in the –1 and +1 positions from the nick causes no significant decrease in the effectiveness of a reaction catalyzed by *Tth* or T4 DNA–ligases [73].

In some cases, the effectiveness of the enzymatic reaction can be less dependent on the bonds in the minor groove and be affected by other interactions. These results were obtained in the studies of mutant forms of A–family DNA–polymerases ( * Taq * , Klenow fragment) at the conserved Gln–Val–His (QVH) motif, which is a part of the С motif [43, 80]. It is known that this motif interacts with the deoxyribose of the 3’–terminal nucleotide of the primer. The histidine residue can then form a hydrogen bond via the minor groove of the duplex. Mutant forms of DNA–polymerases exhibited increased discrimination of substrates bearing mismatches (some of them were as far as 2–4 nucleotides from the enzymatic conversion site [80]), as compared to the wild–type enzymes. The most selective mutants for the Klenow fragment turned out to be PLQ, LVG, LVL and ILL, IVF, CLV for the * Taq* polymerase. Of all the obtained mutants, only PLQ had histidine substituted for glutamine, thus preventing the imidazole ring of the former acting as an electron donor. In most cases, the histidine residue was replaced by amino acids with nonpolar side–chains (leucine, valine, glycine, and phenylalanine) [80]. This suggests that in this case the hydrophobic interactions which stabilize the A–form of the duplex near the site of conversion play a more important role than hydrogen bonds.

The Klenow fragment mutants N675A, R835L, R836A, R841A and N845A have lower selectivity [78]. The exact reason for the effect of these mutations on the selective elongation of the substrate is unclear. The authors suggest that N845 is involved in identifying the “correct” shape of the 3’–terminal base pair. Residues R835, R836 and R841 can interact with the single–strand region of the template strand, thus stabilizing the curve, which can be observed in this DNA fragment. The N675 amino acid interacts with the template at the position where the DNA changes from В – to А –form and can thus be implicated in the stabilization of this conformational disruption.

Substitutions in the amino acid sequence of DNA–ligases can also cause changes in the effectiveness of substrate ligation. Thus, the following mutants of the * E.coli* DNA–ligase with substituted conserved amino–acids in the OB domain (R379A, V383A, I384A and R333A–T334A) were shown to exhibit decreased ligation efficiency, which was no more than 10% of the original activity of the wild–type enzyme [69]. This altered ligation efficiency determined by the functions of the involved amino acid residues is due to the effects on the formation of hydrogen bonds in the minor groove on the template strand’s side of the OH–component (amino acid R379), on the interaction with the carbohydrate backbone of the template strand (R333 and T334), on the formation of a Van–der–Waals interaction network with the base pairs in close proximity to the single–strand break (two pairs in the ОН –component and one in the Р –component), and on the stabilization of the DNA–substrate in A–form (V383 and I384) [69]. DNA–ligases from the *Thermus * family (T АК 16D and АК 16D) were found to have mutants which exhibited increased discrimination of 3’–terminal single mismatches. The mutations D286E, G287A, V289I, and K291R were in the AdD domain [81]; and T599A, in the BRCT domain [82], respectively. In case of the * Tth* ligase, it was found that the use of K294R and K294P mutants led to increased selectivity in the reaction [30]. The causes of these interesting results are unknown. It is however evident that these amino acids are involved at different steps of the enzymatic reaction, and that the increased selectivity is most probably a complex feature involving several steps in the ligation process.

Thus, the selectivity of enzymatic reactions is due to the formation of “correct” bonds in the enzyme–substrate complex. Most probably, the overall effect of these point interactions in the complex creates the basis for discriminating imperfect regions in the dsDNA–substrate.

## Effect of structural disruptions of the DNA substrate on the selectivity of its enzymatic conversion

In the previous section, we reviewed the structure of DNA–dependent enzymes and the complexes they form with the substrate in order to identify the factors affecting the selectivity of enzymatic conversion. This section will review the structural traits of the DNA–complex which can increase the selectivity of the enzymatic reaction. One of the simplest ways to increase enzymatic ligation effectiveness is to use a method based on “modified” probes, which consist of tandems of short oligonucleotides [83–85]. The presence among the ligated components of mini–probes of penta– and even tetra–nucleotides makes these composite complexes less effective as substrates, and their enzymatic selectivity appears to be high [83, 85]. If a tetranucleotide is used as the central part of a three–part tandem, the discrimination factor for any type of mismatch in the region of the substrate complex is more than 300 when using the mesophilic Т 4 DNA–ligase [85]. Such high selectivity of the enzyme is unattainable if the DNA–duplex is formed by oligonucleotides, which are long enough to provide optimal conditions for enzyme binding on the molecule (see [37] for an instance).

## Introduction of an additional single nucleotide mismatch

**Fig. 11:**
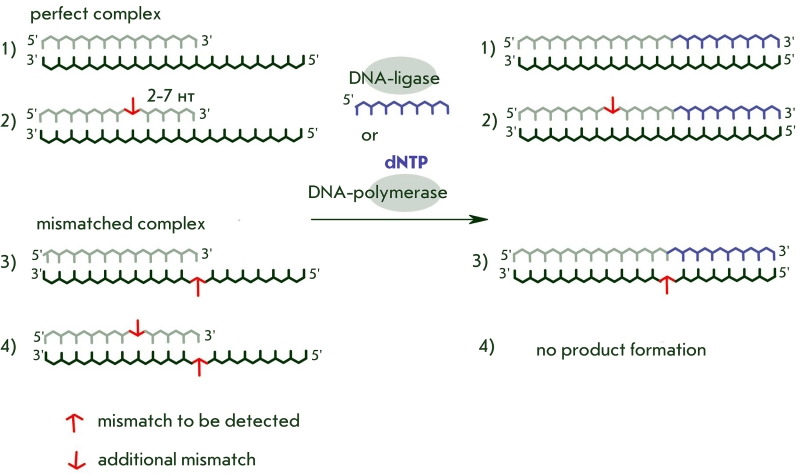
Schematic representation of the process of detection of a non-complementary base in the DNA-substrate during its enzymatic conversion, using an intentional additional mismatch.

Another way to increase the selectivity of enzyme–dependent reactions not involving the use of modified nucleotide analogs is based on the use of DNA–substrates with an intentionally added mismatch next to the polymorphic site to be analyzed. The effectiveness of such an approach was demonstrated for * Taq * DNA–polymerase [14, 86–90] and *Tth* DNA–ligase reactions [30]. This method involves placing the studied mismatch on the 3’–terminus of the elongated oligonucleotide or the ligated ОН –component and the additional mismatch in proximity to the 3’–terminus, specifically in the 2^nd^, 3^rd^ or 4^th^ positions [14, 30, 88–90], and in some cases in the 5^th^ or 7^th^ [86]. In these cases, the “perfect” complex has a single planned noncomplementary pair, while the complex with a mismatch contains two disruptions (Fig. 11).

Comparison of the effectiveness of full–size elongation or ligation product accumulation shows that a single noncomplementary pair does not cause a significant decrease in DNA–substrate conversion, while a double mismatch can lower the end–product yield by a factor of 100 or more [14, 30, 88]. The only exception is seen in case of the 3’–terminal mismatch 3’–T/ ** N ** , when the introduction of a second mismatch only lowers the product yield 5– to 10–fold, and the presence of two neighboring 3’–T/ ** N ** mismatches only lowers the yield 2– to 5–fold, which is not a significant decrease compared to a perfectly paired complex [14]. The effect of the position of the intentional mismatch on the decrease of the false–positive signal during olignucleotide probe processing was also studied [14, 30, 86, 88, 90]. An additional mismatch at any of the above–mentioned positions caused increased discrimination efficiency of the original mismatch. However, an intentional mismatch at the 2^nd^ position did cause a significant decrease in the enzymatic reaction efficiency in several cases. Thus, a *Tth* DNA–ligase reaction exhibited a 175–fold yield reduction even with a complementary pair on the 3’–terminus of the oligonucleotide [30]. On the other hand, a noncomplementary base in the 4^th^ position did not significantly increase selectivity [86, 90]. Of all of the examined positions for introducing an additional mismatch in order to increase the discrimination efficiency of a target mismatch in the enzymatic conversion site in elongation and ligation reactions, the 3^rd^ position from the 3’–terminus proved to be the most effective [30, 88, 90]. In this case, the end–yield of the product which has a mismatched pair on the 3’–terminus was no more than 5 % [90]. The introduction of an additional mismatch was also effective in increasing the discrimination of mismatch during ligation. The ratio between the initial rates of complex conversion of a substrate with a complementary pair at the 3’–terminus and mismatched pair increased by a factor of 4 [30].

Thus, an intentional mismatch introduced into the DNA–substrate structure in addition to the original single nucleotide substitution causes increased enzyme–dependent reaction selectivity. The observed patterns are probably due to the fact that two closely spaced nucleotide mismatches cause a much larger disruption of the DNA structure at the enzyme recognition site and thus a more significant change of stability and structure of the DNA–helix. This causes a double mismatch to have a much stronger impact on the effectiveness of the enzymatic reaction. However, introduction of additional single nucleotide mutations is probably not a universal method for increasing selectivity, since the nature of the introduced “disruptions” is sequence–specific.

## Oligonucleotide modifications that increase the selectivity of DNA-dependent enzymes

Currently, oligonucleotides carrying modified bases or with an altered carbohydrate–phosphate backbone occupy a distinct niche in DNA hybridization probes design. Some modifications (PNA peptydylnucleic acids [91;], LNA and ENA “locked” nucleic acids [92, 93]) increase the stability of the modified complexes, which can be used to increase the accuracy of DNA analysis at the level of hybrid complex formation. Other modifications (N4–alkylcytosine [94], 5–methyl– and 5–(1–propargyl)uracyl [95]) can equalize the hybridizational characteristics of complexes with a different nucleotide content, which is important during a parallel analysis of different DNA sequences. It is worth noting that not all oligonucleotide modifications are compatible with DNA–dependent enzymes, since the introduction of these modifications can disrupt the protein–nucleic interactions which are needed for effective enzymatic catalysis. Nevertheless, introduction of certain nucleotide analogs could become a method for increasing the selectivity of enzymes towards mismatches in modified DNA–duplexes (Table 3).

## Modification of heterocyclic bases

One of the modifications used is a synthetic analog of a deoxyribonucleotide, which bears a universal 3–nitropyrolle base. This base is named universal because it can form pairs with all the natural bases thanks to its small size, which is comparable to that of the natural bases, and the ability it retains to take part in stacking interactions. The effect of this analog on the selectivity of the *Tth* DNA–ligase [30] was studied by introducing it into the 3^rd^ position from the pair to be analyzed, which was placed on the 3’–terminus of the ОН –component. The choice of the position was based on data on increased selectivity upon introduction of an additional mismatch, which was found to be optimal in the –3 position from the enzymatic conversion site. The presence of the nucleotide analog caused a 9–fold selectivity increase of the *Tth* DNA–ligase, which is 2.5–fold more than the increased selectivity effect seen upon the introduction of an additional mismatch based on canonic bases. * Taq * polymerase also exhibited decreased formation of PCR products during the use of a single mismatch and a primer with a 3–nitropyrolle, as compared with a normal primer [96]. The unpredictable binding of oligonucleotides bearing such a modification with the DNA–template is a disadvantage of this approach.

Another nucleotide analog, which can increase mismatch discrimination in *Tth* DNA–ligase reactions, is a 4–nitroimidazole deoxyribonucleotide. When introduced into the probe, it can form a pair with the guanine base, which is less stable than the native C/G–pair [97]. The use of probes with at least 2 such modified analogs lowers the ratio between the ligation products of the mismatched and “perfect” substrates by 15% (average value) as compared to the use of native oligonucleotides [97].

The effect of the above–mentioned modifications is based on the same principle as the introduction of an additional mismatch close to the polymorphism to be detected. The addition of modifications causes destabilization of the DNA–duplex, which can lower the efficiency of the enzymatic conversion only if an imperfect pair is present in the duplex structure. On the other hand, other studies demonstrate that modifications such as 7–deazaguanine and inosine lower the stability of duplexes but do not increase the selectivity of *Tth* DNA–ligase when introduced into a DNA–substrate with a mismatch [97]. This is probably due to the specific steric complications in which these modifications are involved.

Overall, even though the analog nucleotides modified at their heterocyclic bases can form hydrogen bonds with the native nucleotides, it is evident that these bonds are different from Watson–Crick bonds. This may be one of the reasons for the disruption of the DNA–helix. The use of these analogs in the hybridization analysis of oligonucleotides with a modified carbohydrate–phosphate backbone may prove to be more effective, since this approach does not involve the part of the nucleotide which forms complementary interactions with the NA–template.

## Modifications of the carbohydrate residue

Such derivative oligonucleotides involve oligomers, which contain modified bicyclic RNA–like monomers with 2’–O, 4’–C methylene and 2’–O, 4’–C ethylene links, LNA (Locked Nucleic Acid) [98–102], and ENA (Ethylene Nucleic Acid) [103], respectively.

LNA– and ENA–containing oligonucleotides exhibit an increased affinity to the complementary NA–template, and this can be used for detecting mismatches in a PCR reaction, since the complex forms with higher specificity [93]. Primers with a single LNA–modification at the exact 3’–terminus or in the last but one position, or with an ENA–analog in the third position do not lower the effectiveness of elongation of modified primers by * Taq* DNA–polymerase if the DNA–substrate is perfect, but they facilitate discrimination of 3’–terminal mismatches if the forming DNA–complex is imperfect [98–103]. The introduction of modifications into the 3’–terminal region of the oligonucleotide lowers the yield of the PCR reaction in the presence of “difficult to detect” mismatches (C/A, T/G, G/T) by at least 50% as compared to the use of a native primer [98, 99, 103]. The reason for such altered selectivity of oligonucleotides with bicyclic analogs may be due to the altered behavior of the DNA–substrate in the active site of the enzyme. The presence of a locked “link” causes the fixation of the nucleotide’s ribose in the 3’–endoconformation [104], which is characteristic of nucleotides in an A–form dsDNA–helix. Also, the modified nucleotide itself shows less conformational flexibility. Thus, LNA– and ENA–modifications facilitate the locking of the DNA–substrate in A–form, which has a positive impact on the formation of protein–nucleic interactions in the minor groove of the DNA–substrate.

Another type of modification of the carbohydrate residue which increases the selectivity of DNA–polymerase activity is the use of C4’–alkylated thymine nucleotides **T_R_** [105–108]. These modifications, which cause the formation of atypical groups in the minor groove of the DNA–complex, were first tested for effectiveness of the elongation of an oligonucleotide modified at the 3’–terminal position. Such reactions depend on the size of the alkyl residue and the type of DNA–polymerase used. * Thermococcus litoralis * ( * Vent* ) archaeal polymerase was found to elongate the modified oligonucleotide even when a large vinyl residue was introduced, while the archaeal * Pyrococcus furiosus * ( *Pfu * ) polymerase could only elongate a primer that bore only the smallest available methyl side chain. * Taq * polymerase could not elongate modified oligonucleotides. The authors suggest that the inability to elongate a primer with a large 3’–terminal nucleotide was due to steric restrictions and could be explained for each individual polymerase by analyzing the size and shape of its active site [107]. С 4’–alkyldeoxyribothymidine increases mismatch discrimination both when it is one of the mismatched nucleotides, but also if it is the second or third pair from the primer terminus [105]. Probes with a modified monomer exhibit increased mismatch discrimination, which rises in direct proportion to the size of the side–chain at the С 4’–position of deoxyribose. The selectivity of the * Vent* polymerase catalyzed primer elongation increases in the following sequence: ** T ** < **T_M_**_ e _ < ** T_Ey_** < **T_Vi_** (See chemical structures in Table. 3).

The Δ С _ t _ value which equals the difference in the number of the threshold cycle during PCR comparison is 8.5 cycles for the formation of a perfect and 3’–terminally mismatched DNA–complex when using a modified ( С 4’–ethyl) primer. This value is close to zero when using a nonmodified primer [107]. This increased selectivity of the PCR reaction is evident even when the mismatches are removed up to 4 bp from the conversion site [106]. The authors note that introduction of such nonpolar side–chains into oligonucleotide primers does not destabilize the DNA–complexes, and the PCR characteristics of these duplexes are not affected by buffering conditions or the nucleotide composition of the formed duplexes. Thus, the observed increased selectivity of the DNA–polymerase is caused by a disruption of the network of enzyme/substrate interactions due to the exposure of С 4’–side–chains into the minor groove.

The authors took their research further and decided to use combinations of modifications. The 3’–terminus of the oligonucleotide primer was modified with a thymidine analog bearing a 4’– С –methoxymethylene residue in its deoxyribose, as well as a thiol group in the С 2 or С 4 position of the thymidine base [109]. Reactions with the * Vent* DNA–polymerase and primers with a single modification exhibited increased discrimination only with the use of a carbohydrate–substituted oligonucleotide ( ** T_OMe_** , Δ С _ t _ = 9). The presence of a thiol group in the C2 position of the base ( **^ 2S ^ T ** ) caused only a slight increase in PCR selectivity ( **^ 2S ^ T ** , Δ С _ t _ = 3), and it did not change it at all at the С 4 position ( **^ 4S ^ T ** , Δ С _ t _ ≈ 0). However, the simultaneous presence of the methoxymethylene residue and modification of the heterocyclic base caused a dramatic increase in the discriminative ability of the DNA–polymerase. The Δ С _ t _ values for dually modified primers **^ 2S ^ T_OMe_ and ^4S^ T_OMe_** were 12 and 19 cycles, respectively.

Thus, it was demonstrated that combined use of various types of modifications can yield a high level of DNA–substrate mismatch discrimination by enzymes. Most probably, the DNA–complexes exhibit such characteristics because of the steric difficulties that occur in the case of a side–chain in the ribose residue, and in the case of an added thiol group, both of which have their most dramatic effect when placed in the opposite strand from the nucleotide analog of the noncomplementary base. On the other hand, the 3’–terminal nucleotide primer is highly dependent on the formation of hydrophobic bonds during the formation of an enzyme–substrate complex (discussed in the previous section). Nonpolar side–chains could probably facilitate such contacts.

**Table 3:** Oligonucleotide modifications that promote the selective activity of DNA-dependent enzymes

Heterocyclic base modifications 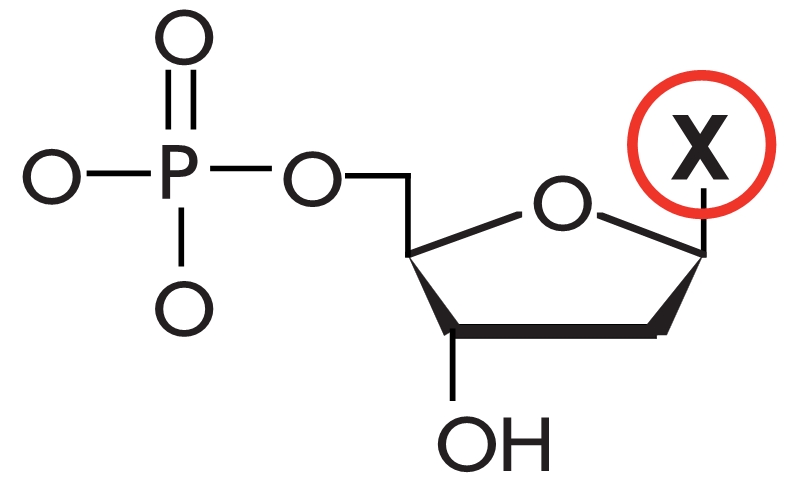	Carbohydrate backbone modifications 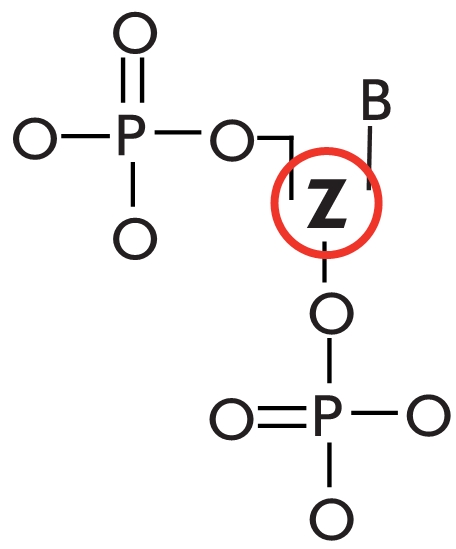	Internucleotide phosphate modifications 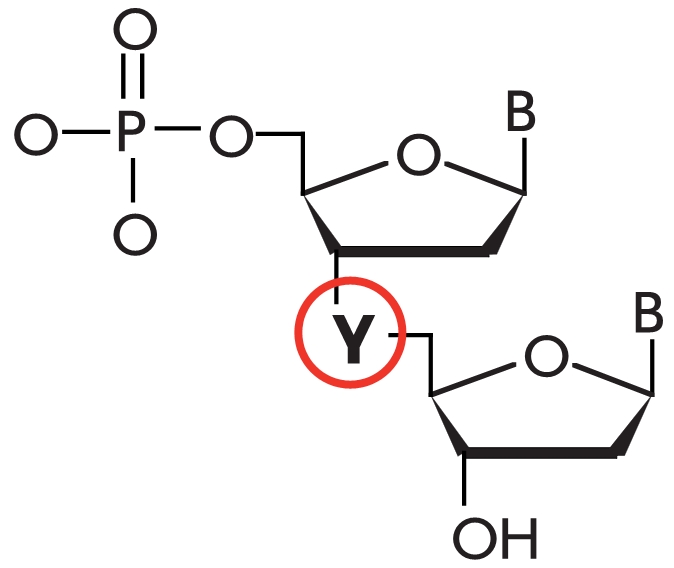
3-nitropyroll [30, 96] 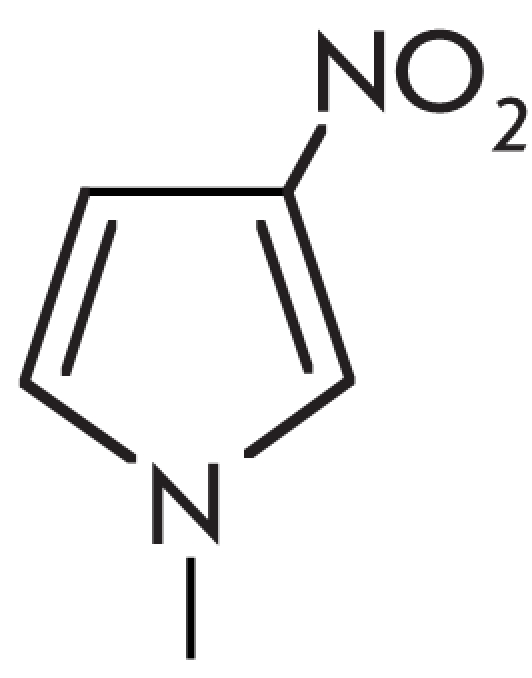 4-nitroimidazole [97] 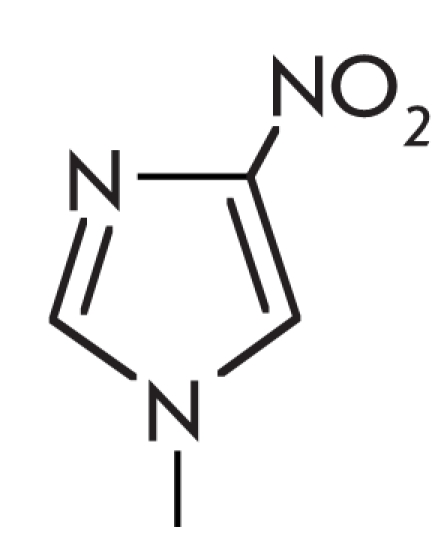 С2, С4-thiothymidine [109] 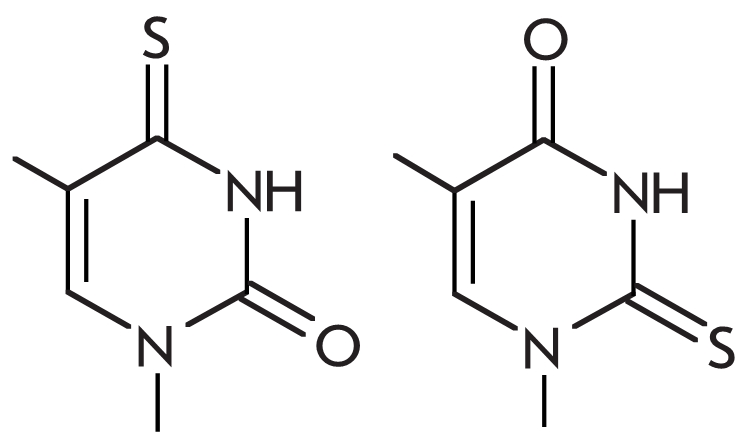	2'-O, 4'-C methyleneribose (LNA) [98−102] 2'-O, 4'-C ethyleneribose (ENA) [103] 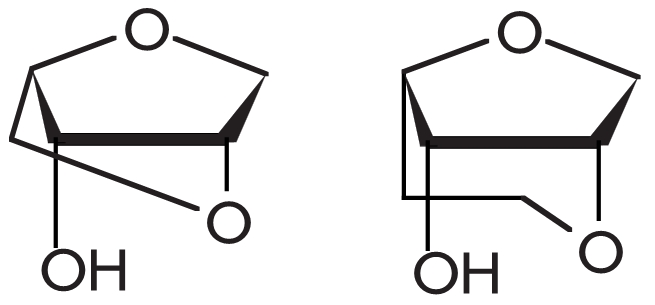 С4'-alkylribose [105−109] 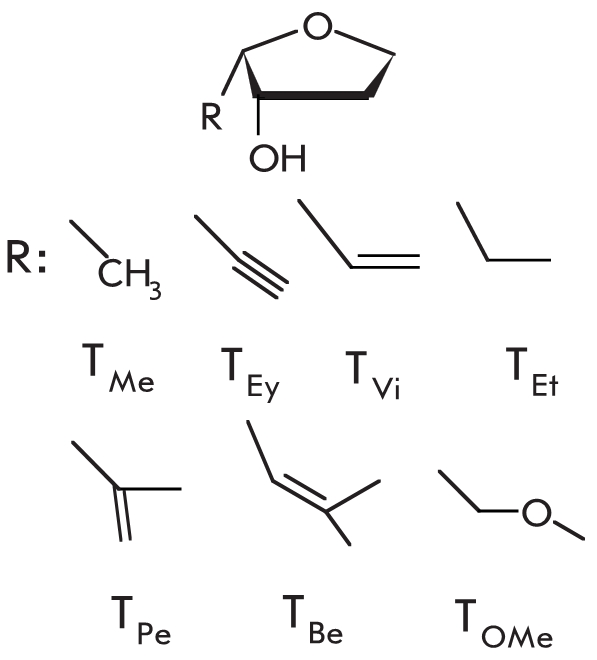	thiophosphate [110−112] 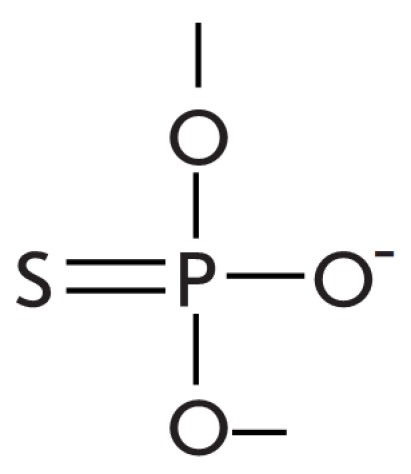 oligoethyleneglycol [13, 113] 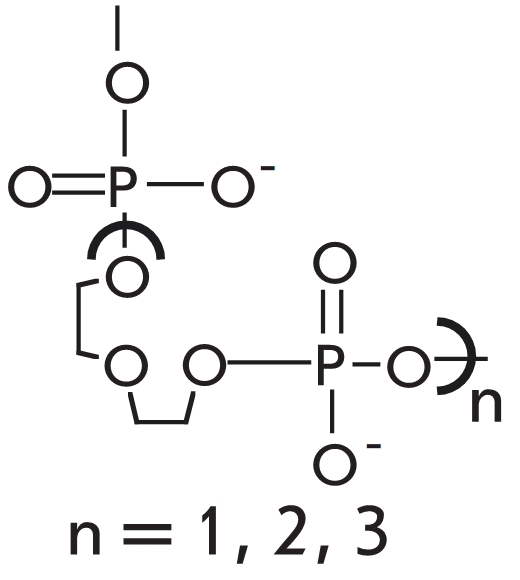 oligomethylenediol [13, 113] 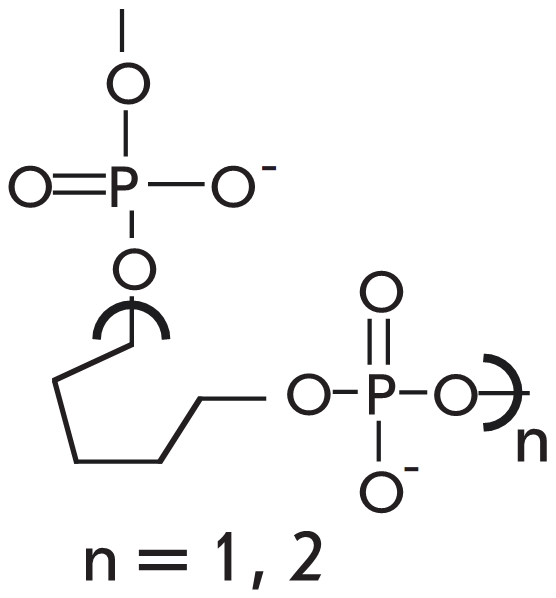

## Modifications of the internucleotide phosphodiester residue

Increased selectivity of DNA–polymerases was demonstrated during the use of oligonucleotide primers modified at the internucleotide phosphodiester residue [110–112]. Substitution of the native phosphate groups, located between the first and the second nucleosides at the 3’–terminus of the primer, with thiophosphates increased the 3’–terminal mismatch discrimination efficiency of * Vent * and *Pfu * DNA–polymerases [110–112]. Such modifications did not alter the stability of lengthy DNA–complexes, but their presence increased the discrimination of nucleotide mismatches, single or multiplex, and even those located at a distance from the enzymatic conversion site. The DNA–polymerases showed no detectable elongation of the modified oligonucleotide, even when the mismatches were located up to 8 nucleotides from the 3’–terminus of the primer [111]. Notably, according to the data presented above, DNA–polymerases form tight interactions with the carbohydrate–phosphate backbone of the DNA helix up to the above–mentioned position in the primer strand. However, the authors also noted that for the conditions of the enzymatic reaction to be as stringent as possible, reactions using phosphothioate analog oligonucleotides had to be performed with DNA–polymerases that possesed exonuclease proofreading activity and the conditions for allele–specific PCR had to be adjusted [112].

The use of oligonucleotide probes bearing nonnucleotide insertions into the carbohydrate–phosphate backbone also exhibited increased single mismatch discrimination efficiency of DNA–substrates during enzymatic ligation using T4 phage DNA–ligase, and during elongation by * Taq * DNA–polymerase as compared to the use of native DNA primers [13, 113]. The presence of insertions based on phosphodiesters of oligoethyleneglycol and oligomethylene diols inside the enzyme–binding site on the DNA–substrate or at its border caused a significant increase in the selectivity of the modified probe conversion. Enzymes could discriminate mismatches that were located at a distance from the enzymatic conversion site if the complexes bore this nonnucleotide loop. The mismatch discrimination factors for reactions with mismatches located six nucleotides away from the conversion site and an oligonucletide probe with an insertion at the 6^th^ position amounted to up to 8 for ligation and 12 for elongation. The use of a native probe in such a complex did not provide efficient discrimination of a single mismatch in the duplex, and the mismatch discrimination factor was 2.9 and 1.2 for ligation and elongation, respectively [13].

The authors note that the presence of a nonnucleotide loop in the substrate complex is a sort of disruption of the substrate structure, which lowers the efficiency of enzymatic catalysis in any case, but the presence of an additional disruption, as in case of a mismatch, can considerably increase the probability of its discrimination and thus explain the observed increase in the selectivity of allele–specific enzymatic reactions.

Thus, introduction of “disruptive” elements into the structure of the DNA–substrate of enzymatic reactions can sometimes help achieve the desired accuracy in mismatch discrimination. Artificial modifications of the DNA–complex structure can increase the selectivity of DNA–ligases and DNA–polymerases, although a lowered efficiency of enzymatic conversion is also observed. In the future, modifications that affect the fidelity of the enzymatic reaction at the level of stabilization of the correct conformation of the reactive complex and facilitate the formation of vital enzyme–substrate interactions may prove to be the most effective way to increase selectivity.

## CONCLUSIONS

The data reviewed in this paper prove that the problem of achieving high selectivity in the enzymatic conversion of oligonucleotide probes during nucleotide polymorphism analysis in DNA is an issue depending on multiple factors. It is safe to assume that a universal analysis scheme which allows an unequivocal discrimination of any nucleotide variation in DNA and which uses the discussed analytic approaches has yet to be devised. The choice of a DNA analysis scheme requires a complex design of the components of the analytical procedure, which factors in the “two sides of the same medal.” Firstly, it is the specifics of substrate complex recognition by the DNA–processing enzyme, and secondly, the structural characteristics of the DNA–substrate which is formed by a molecular probe, based on an oligonucleotide or its derivative. This review summarizes the most relevant facts that characterize the peculiarities of nucleotide polymorphism analysis of DNA using DNA–ligases and DNA–polymerases. The data presented reveal the fundamental principles of selective oligonucleotide probe conversion during enzymatic DNA–analysis and also point out the most promising recent developments in this field of research. Our analysis of the available data shows that, despite the large amount of studies reviewed in this paper, the problem of achieving selectivity in probe conversion remains unresolved and undoubtedly requires further research.

## ABBREVIATIONS

PCR: polymerase chain reaction
NA: nucleic acid
AdD: nucleotidyltransferase domain of DNA ligases
OB: oligonucleotide/oligosaccharide binding domain of DNA ligases
DBD: DNA binding domain of DNA ligases
HhH: motif of DNA ligases helix-hairpin-helix
Zn: zinc-fingers
BRCT: C-terminal domain of DNA ligases
PNA: Peptide Nucleic Acids
LNA: Locked Nucleic Acid
ENA: Ethylene Nucleic Acid
dNTP: deoxyribonucleosidetriphosphate
PPi: inorganic pyrophosphate
mc: main chain of protein backbone
